# Why Financial Literacy Matters for Dental Students: Reducing Debt Account Aversion to Improve Debt Management

**DOI:** 10.1002/jdd.70211

**Published:** 2026-04-10

**Authors:** Junsu Park, Jungjoon Ihm

**Affiliations:** ^1^ Division of Convergence Management Department of Business Administration Dongguk University WISE Gyeongju Republic of Korea; ^2^ Dental Research Institute School of Dentistry Seoul National University Seoul Republic of Korea

**Keywords:** debt account aversion, debt management, dental students, financial education, financial literacy

## Abstract

**Objectives:**

In South Korea, dental graduates frequently incur substantial debt to establish private practices; however, formal financial education is rarely incorporated into undergraduate dental curricula. This study examines whether undergraduate dental students exhibit debt account aversion (DAA)—a cognitive bias in which small debts are prioritized over those with high interest rates—and whether financial literacy predicts susceptibility to this bias.

**Methods:**

Seventy‐one undergraduate dental students from a top‐ranked South Korean university completed a standardized 15‐item financial literacy test followed by a 25‐round debt management game. In the game, the participants repaid six hypothetical debts with varying sizes and interest rates to minimize the total remaining debt. The total debt after 25 rounds served as the primary outcome. To reflect individual variability in DAA, the study formulated a DAA index to quantify the extent of bias by measuring the proportion of repayments allocated to the smallest open debt account.

**Results:**

The participants ended the game with significantly higher remaining debt than the financially optimal benchmark, reflecting partial adherence to DAA‐like repayment preferences. Financial literacy was negatively associated with DAA and total debt, whereas DAA was positively associated with total debt. Mediation analysis supported the direct and indirect effects of financial literacy on total debt via reduced DAA.

**Conclusion:**

The presence of DAA among dental students highlights the need for the integration of financial education into dental curricula. Improving financial literacy may help reduce this bias and enhance debt management, sustainability of practice, and quality of patient care.

## 1 Introduction

Dental students typically face a critical career decision: whether to seek employment in large‐scale medical institutions or establish private practices. In South Korea, the majority of dental clinics are operated as solo practices [1, 2], requiring dentists to independently manage financial risks associated with rising startup costs and intensifying market competition [3]. These conditions highlight the importance of individual‐level financial decision‐making and debt management competencies in modern dental careers. Consequently, financing strategies have become a major concern, particularly because, under the Medical Service Act^1^, direct investment from government or government‐affiliated institutions is unavailable for practice startups [4]. Given these constraints, startup funds are typically raised through personal capital or bank loans, thus making loans the primary source of external financing [5].

Thus, solo practices can achieve long‐term sustainability only when their owners possess robust debt management competencies, given their heavy reliance on external financing. As a result, newly graduated dentists are often required to manage multiple debt accounts on their own, making effective individual debt management a core professional competency. Under these conditions, cognitive biases in individual debt repayment decisions may lead to inefficient loan prioritization and excessive interest payments, increasing financial vulnerability at the individual level. Therefore, debt management should be regarded as a core managerial competency that is essential for the success of solo dental practices. Research on behavioral economics, which demonstrates that young adults frequently face difficulty in financial decision‐making—including debt repayment strategies—due to cognitive biases, further underscores the importance of cultivating these skills during dental education [6]. For instance, Amar et al. [6] examined the debt management behavior of undergraduate students in the United States using a 25‐round repayment game that features six debts with varying amounts and interest rates. The findings revealed that when asked to minimize total debt across 25 rounds, the participants frequently exhibited debt account aversion (DAA)—a bias that tends to prioritize payments of small debts instead of those with high interest rates—ultimately leading to higher interest costs and increased financial losses. In the context of dental practice establishment, such biased decision‐making could lead to inefficient loan repayment and heightened financial vulnerability at the individual level, even before entering real‐world practice environments. Against this background, the current study intends to determine whether dental students exhibit DAA in terms of debt repayment decisions.

Despite the negative consequences of DAA, relatively less is known about the factors that influence one's susceptibility to this bias. Identifying such factors is essential for the development of targeted interventions to improve financial decision‐making among future professionals, including dental students. This study proposes financial literacy as a key predictor of DAA. Financial literacy refers to an individual's ability to apply financial knowledge and skills to the effective management of financial resources and to support long‐term financial well‐being [7]. Prior studies proposed that college students with low levels of financial literacy are more likely to reach suboptimal decisions in savings, borrowing, and investing [8]. Other studies observed similar patterns in older adult samples: people who struggle with interest rate calculations tend to accumulate more debt but less wealth [9], while those who underestimate the effects of compound interest are more likely to face difficulty in debt repayment [10].

This concern is not limited to western contexts; recent national data illustrate the financial literacy challenges faced by South Korean adults. The National Financial Literacy Survey reported that although South Korean adults generally understand the basic concept of interest, their comprehension level of compound interest calculations remains notably low [11]. In particular, individuals aged in their 20s obtained scores less than the national average for overall financial literacy. This finding indicates that undergraduate dental students—a subgroup within this age range—may also struggle to make rational debt repayment decisions when managing multiple loans. However, this potential vulnerability is unlikely to be mitigated through formal education, as South Korean dental curricula provide limited opportunities for financial training. Most dental schools in South Korea lack dedicated courses on financial management or personal finance in their core curricula. Instead, limited aspects of practice management—such as accounting, labor administration, and insurance claims—are briefly addressed in senior‐year courses like Dental Healthcare Management, which primarily focus on infection control. This structural lack of financial education likely heightens students’ susceptibility to cognitive biases in debt repayment, thereby increasing the risk of adopting suboptimal strategies such as DAA.

Taken together, this study aims to achieve two objectives: (1) to investigate whether undergraduate dental students in South Korea exhibit DAA in terms of debt repayment decisions and (2) to examine whether financial literacy is a significant predictor of DAA. By addressing these objectives, the present study extends prior research on financial literacy and student debt in several important ways [12, 13, 14]. Whereas existing studies among health professions students have primarily relied on self‐reported surveys to assess financial knowledge, debt burden, stress, or perceived educational needs, the current study adopts an experimental behavioral approach to directly observe debt repayment decisions under controlled conditions.

Specifically, the study employs a validated multi‐round debt management game [6] to examine DAA as a cognitive bias in repayment prioritization, thereby shifting the focus from debt as a static outcome to debt management as a dynamic decision‐making process. In addition, this study introduces a novel quantitative DAA index that captures individual‐level variability in repayment behavior across repeated decisions, enabling a mediation analysis to clarify the behavioral pathway through which financial literacy influences debt outcomes. Together, these contributions highlight the distinctiveness of the present research and underscore its relevance for integrating behaviorally informed financial education into dental curricula. These efforts are particularly relevant given the substantial financial responsibilities that future dentists must assume during the early stages of their careers.

## 2 Materials and Methods

### 2.1 Participants

We used a convenience sample of undergraduate dental students recruited from South Korea's top‐ranked university, which admits only 45 students per year. The size of the eligible participant pool was limited due to factors such as mandatory military service, health‐related leaves of absence, and voluntary participation. A total of 71 undergraduate dental students (men: 39, *M*_age_ = 20.63, SD = 2.66) participated in the study. The sample included 29 first‐year (40.8%), 32 second‐year (45.1%), and 10 third‐year (14.1%) students; no fourth‐year students were included. Fourth‐year students were excluded because their curriculum includes a compulsory course, Understanding Business Administration for Dentists, which covers general management topics such as budgeting, human resource management, service marketing, and accounting. Although this course does not provide formal instruction in financial literacy or debt management, it introduces certain aspects of practice management that could influence financial decision‐making. Accordingly, to ensure that all participants represented students without prior exposure to structured financial education, only first‐ to third‐year students were recruited for the present study.

These first‐ to third‐year dental students represent a group with exceptionally high academic achievement, as admission to this university's dental school requires top scores on the national university entrance examination. Demonstrating the presence of DAA—a cognitive bias previously identified among US undergraduates [6]—even within this academically elite sample would offer robust evidence for the generalizability of DAA. Moreover, the persistence of this bias among top‐achieving students would further underscore the need to incorporate formal financial education into dental curricula to support more rational and sustainable debt management behaviors. The institutional review board approved all procedures (IRB approval #S‐D20210009), and written informed consent was obtained from the participants.

### 2.2 Procedure

The study was conducted in January 2023 in a laboratory at the Graduate School of Dentistry, and data were collected in a controlled setting, where participants individually completed the online tasks while seated at laptop‐equipped desks. Using a provided link, they first completed a self‐report measure of financial literacy, followed by a debt management game developed by Amar et al. [6] and, finally, a brief demographic questionnaire.

### 2.3 Measures

#### 2.3.1 Financial Literacy

Financial literacy was measured using 15 of the 16 questions (see Supporting Information S1) originally developed by Van Rooij et al., as part of two modules in the De Nederlandsche Bank (DNB) Household Survey [15, 16]. These modules were designed to capture variation in individuals’ financial knowledge across levels of difficulty. The basic module (5 items) assesses numeracy and understanding of concepts such as inflation, interest rates, compound interest, and the time value of money, while the advanced module (11 items) evaluates knowledge of complex financial instruments—including stocks, bonds, and mutual funds—and principles of risk diversification and the risk‐return trade‐off.

Although internal consistency statistics were not reported in the original studies [15, 16]—reflecting the heterogeneous and knowledge‐based nature^2^ of the financial literacy measure—this instrument has been extensively validated and widely adopted and adapted across diverse international studies [21, 22, 23]. Recent analyses using DNB Household Survey data (2005–2018) further confirm its robustness over time and its predictive validity for financial outcomes such as retirement planning, wealth accumulation, and stock market participation [23].

One bond‐related question (Item 9 in the original 16‐item set) was removed to prevent conceptual redundancy and maintain balance within the measure. Several items in the advanced literacy module already addressed bond‐related concepts—such as distinctions between stocks and bonds, long‐term bond risk, and the relationship between bond prices and interest rates—resulting in conceptual overlap. Also, prior research has shown that bond‐related questions tend to be the most difficult for respondents [15], which likely makes them even more challenging for undergraduate dental students with limited exposure to formal financial education. This adjustment aimed to provide a more balanced representation of financial concepts across the questionnaire. Each correct answer takes a value of 1, while incorrect and “don't know” responses take a value of 0. The total scores range from 0 to 15. Higher scores indicate greater overall knowledge of various financial topics.

#### 2.3.2 Debt Management Game

After the literacy assessment, the participants played the game, using the same scenario as employed in Amar et al. [6]. In this scenario, they were hypothetically assigned six debts (A–F), which varied in size and annual interest rate (Table 1).

**TABLE 1:** Annual interest rate and initial amount of each debt.

Debt	Annual interest rate (%)	Initial amount ($)
A	2.50	3000
B	2.00	8000
C	3.50	11,000
D	3.25	13,000
E	3.75	52,000
F	4.00	60,000

The game consisted of 25 rounds, each round represented a year. At the beginning of each round, the participants received a fixed income of $5000. Additional bonuses were given in rounds 6 ($20,000), 12 ($15,000), and 19 ($40,000). For each round, the participants were required to develop a repayment plan by choosing which debt(s) to pay and typing in the amount to allocate to each account. The full budget available for the round needed to be used for debt repayment before proceeding to the next round. After each round, the program provided an updated balance for each debt account through a graphical summary of repayment progress. After 25 rounds, the total debt was automatically calculated by subtracting the amount of cash left in the checking account (always zero^3^) from the sum of the remaining balance of each debt account. Total debt was used as the main dependent variable in the analyses (for a sample screenshot, see Supporting Information S2).

A *financially optimal player* (FOP) would spend all of the available budget per round by paying off debts in a descending order of interest rate. For example, the player would first repay the debt in Account F (4% interest rate), followed by the debt in Account E (3.75% interest rate), and so on, until the game ended. By doing so, the debts in Accounts D, A, and B would remain after the final round. Nevertheless, this strategy would be optimal, because it would result in the smallest possible total debt: $29,428 (see Supporting Information S3). Conversely, a *debt‐account‐averse player* may prioritize the repayment of debts in an increasing order of amount. For example, they would first pay off the debt in Account A ($3000), then the debt in Account B ($8000), and so on, until the end of the game. This strategy would be suboptimal, because it will leave the participant with a total debt (in Account F) of $47,861, which is nearly double that of the FOP (see Supporting Information S4).

To encourage participants to minimize the final debt, the game was designed to be incentive compatible. In addition to a $4 show‐up fee, the participants were eligible for a performance‐based bonus as follows: $4, $3, $2, and $1 for debts of $30,000 or less, $30,001–$35,000, $35,001–$40,000, and more than $40,000, respectively. Nevertheless, after the study and a full debriefing, all participants received a gift card worth $8 regardless of their total debt at the end of the game. This approach aimed to prevent potential negative effects on participants’ self‐efficacy that might stem from smaller rewards being perceived as indicative of poor financial ability, while preserving their motivation to minimize debt within the task.

#### 2.3.3 DAA Index

Although Amar et al. [6] demonstrated the presence of DAA through behavioral patterns (e.g., by examining the round in which each debt was fully repaid), they did not provide a quantitative measure to assess the degree of this bias at the individual level. To address this limitation, the present study developed a novel DAA index to capture the extent to which participants exhibited DAA during the game. This index allows for a more fine‐grained analysis of individual variability in DAA and facilitates the examination of how financial literacy predicts the magnitude of DAA and, consequently, debt outcomes (i.e., total debt). In this study, DAA was operationalized as the allocation of any amount of money to the smallest open debt account. For each of the 25 rounds, we identified whether participants allocated any portion of their available budget—including the fixed $5000 income and three bonus payments ($20,000, $15,000, and $40,000 in Rounds 6, 12, and 19, respectively)—to the smallest remaining debt. The proportion of these non‐optimal allocations relative to the total available budget for that round was calculated. For example, in Round 1, if a participant allocated the entire $5000 income to the smallest open debt (Debt A: $3000) and the next‐smallest debt (Debt B: $2000), then the DAA score for that round would be 1.0 ($5000/$5000), indicating complete DAA. If $3000 was allocated to Debt A and the remaining $2000 to other debts (Debts C–F), then the score would be 0.6 ($3000/$5000), indicating a high level of DAA. Conversely, if all funds were allocated to debts apart from the smallest (Debts B–F), then the score would be 0, reflecting the absence of DAA. Notably, if the smallest open debt also carried the highest interest rate in any given round, then any allocation to it was not counted toward DAA, and the bias score for that round was set to 0, because the decision aligned with financially optimal behavior. These proportions were then averaged across the 25 rounds to produce individual DAA scores. Higher DAA scores represented a stronger behavioral tendency to prioritize payments of small debts instead of those with high interest rates.

#### 2.3.4 Control Variables

Based on the responses to the demographic questionnaire, the study included age, gender, and school year as the control variables to account for their potential influence on the total debt. The study controlled for age, because younger individuals tend to carry revolving credit card balances more frequently and manage debt less effectively than older adults, whose comprehension of debt typically improves over time [10]. Gender was included, given documented differences in financial behaviors and attitudes such as risk tolerance and debt management strategies [24, 25]. Age was measured using a continuous scale (in years), while gender (1 = *men*, 2 = *women*) and school year (1 = *freshman*, 2 = *sophomore*, and 3 = *junior*) were set as the categorical variables.

## 3 Results

The main dependent variable was the total debt that remained after completion of the 25 rounds of the game. This value reflects the extent to which each participant repaid the original debt of $147,000. We examined two key questions: (a) whether the participants made financially optimal repayment decisions, and (b) whether financial literacy influenced DAA and, thus, decreased the total debt.

Consistent with Amar et al. [6], 93% of participants exhibited repayment patterns that fell between the DAA and FOP benchmarks. No participant followed a purely DAA‐based strategy, whereas only five adopted a financially optimal strategy. Across all participants, the mean remaining debt was $35,986 (SD = $5163; range = $29,428–$45,896). To test differences between the two theoretical benchmarks, one‐sample *t*‐tests were conducted. The mean remaining debt was significantly higher than that of the financially optimal benchmark ($29,428; *t*(70) = 10.70, *p* = 10^−16^, *d* = 1.27) and significantly lower than that of the DAA benchmark ($47,861; *t*(70) = −19.38, *p* = 10^−30^, *d* = 2.30). These results suggest that dental students exhibited bounded optimality in their repayment decisions, demonstrating behaviors that were neither fully optimal nor entirely consistent with DAA. This pattern indicates a tendency toward non‐optimal debt management, which may reflect partial adherence to DAA‐like repayment preferences.

To further explore whether this non‐optimal behavior reflected the DAA tendency, we compared repayment patterns to the two benchmarks. Specifically, we analyzed the average round in which the participants fully repaid each debt account^4^ compared to when these accounts were closed under the two benchmarks.

**FIGURE 1:**
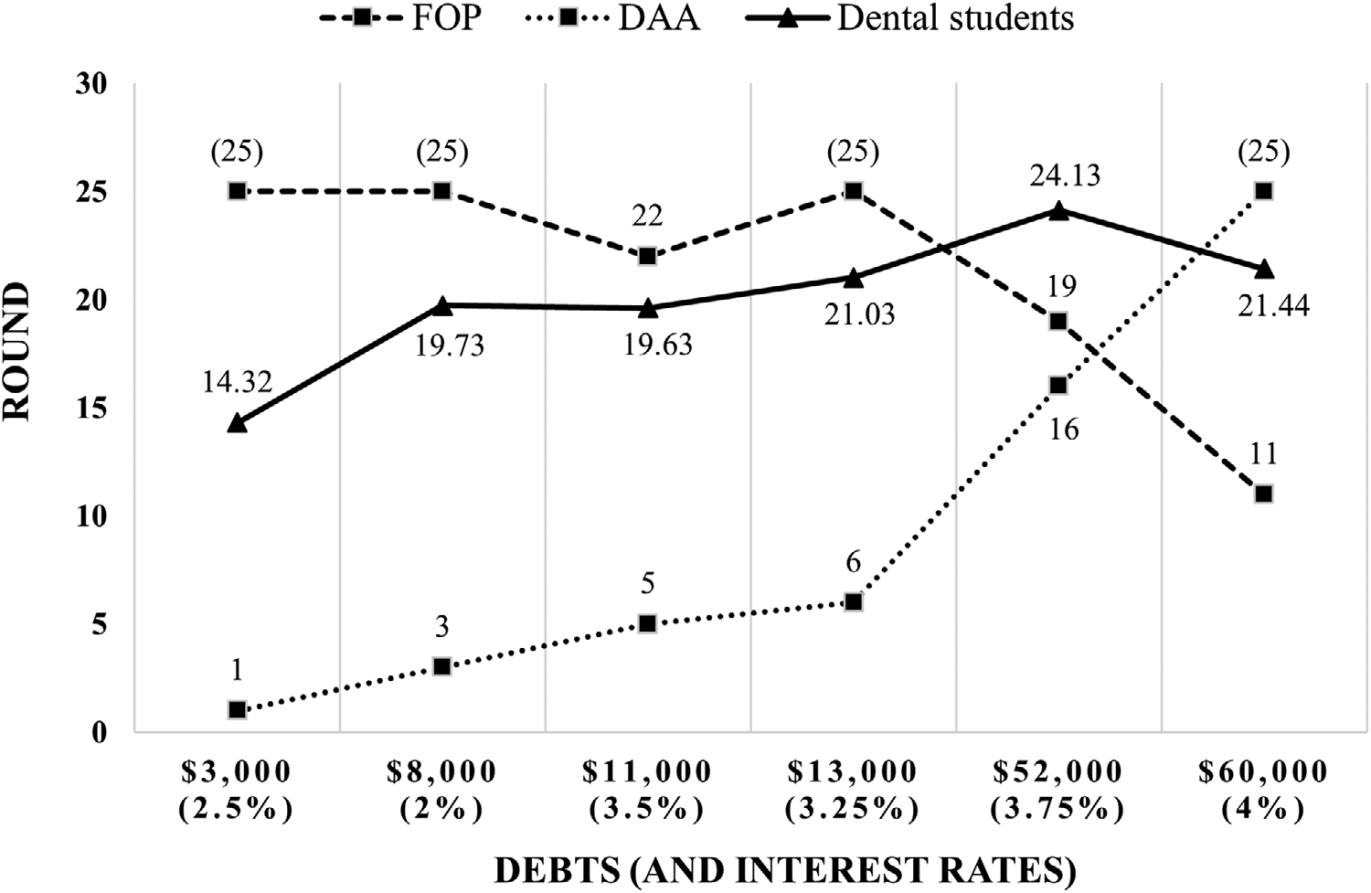
Average round each debt was closed compared with financially optimal and debt‐account‐averse benchmarks. (25) indicates debts that should remain open at the end of the game.

Figure 1 illustrates that the participants tended to repay smaller debt accounts first, despite their lower interest rates. This behavior resulted in a positively sloped repayment trajectory, which closely matches the DAA benchmark, and contrasted with the negatively sloped trajectory of the financially optimal strategy, which prioritizes high interest debts. These findings imply that dental students generally prioritized the repayment of small debts compared with those with high interest rates, which results in financially suboptimal repayment patterns consistent with DAA. This result replicates the DAA originally observed by Amar et al. [6].

Despite the notable findings, comparing the rounds in which participants fully repaid each debt with those predicted by the two benchmarks did not accurately capture the extent of bias at the individual level. To further quantify individual differences in repayment behavior, a DAA index was computed for each participant based on their allocation patterns across 25 rounds (see Methods for calculation details). We hypothesized that participants with higher scores for financial literacy would exhibit lower levels of DAA and, consequently, have lower total remaining debts by the end of the game. Table 2 presents the correlations among the variables.

**TABLE 2:** Correlations among study variables.

Measure	Mean	SD	1	2	3	4	5
Control variables							
(1) Gender	0.55	0.50	—				
(2) Age	20.63	2.66	0.03	—			
(3) Academic year	1.73	0.70	−0.21	0.37^**^			
Predictors							
(4) Financial literacy	10.62	1.94	0.05	−0.11	−0.10		
(5) DAA	0.07	0.11	−0.27^*^	0.13	0.17	−0.37^**^	
Criterion variables							
(6) Total debt	$35,986	$5163	−0.26^*^	0.13	0.13	−0.55^**^	0.77^**^

*Note*: Gender was coded as men = 1 and women = 0.

* *p* < 0.05;

** *p* < 0.01.

As expected, the total remaining debt was significantly correlated with financial literacy (*r* = −0.55, *p* < 0.01) and DAA (*r* = 0.77, *p* < 0.01). In addition, the two predictors were significantly correlated (*r* = −0.37, *p* < 0.01). These correlations indicate that higher levels of financial literacy may reduce the total remaining debt, partially by mitigating DAA. To test this hypothesis, we conducted a mediation analysis using SPSS PROCESS macro (Model 4) [26] and set financial literacy as the predictor, DAA as the mediator, and total remaining debt as the outcome variable, while controlling for gender, age, and academic year. Table 3 presents the results.

**TABLE 3:** The mediation effect of DAA in the relationship between financial literacy and total debt.

	Effect	SE	*t*	*p*	95% LLCI, ULCI
Indirect effect of *X* on *Y*	−539.14	168.93			(−879.78, −216.56)
Direct effect of *X* on *Y*	−830.89	201.51	−4.12	0.0001	(−1233.46, −428.32)
Total effect of *X* on *Y*	−1370.04	260.02	−5.23	0.0000	(−1893.33, −846.74)

Abbreviations: LLCI = lower‐limit confidence interval; SE = standardized error; ULCI = upper‐limit confidence interval.

**FIGURE 2:**
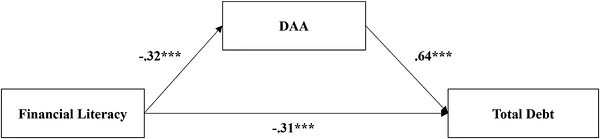
Mediating model with standardized path coefficients; DAA is the mediating variable in the relationship between financial literacy and total debt. *p < 0.05; **p < 0.01; ***p < 0.001.

Table 3 depicts a significant direct and indirect effect of financial literacy on total remaining debt through DAA after controlling for gender, age, and academic year. Specifically, financial literacy was negatively associated with DAA (*B* = −0.02, SE = 0.01, *p* < 0.01), whereas DAA was positively associated with total remaining debt (*B* = 31032.46, SE = 3985.99, *p* < 0.001). The direct effect of financial literacy on total remaining debt remained significant after accounting for DAA (*B* = −1370.04, SE = 262.02, *p* < 0.001), which indicates that financial literacy influenced total remaining debt directly and indirectly through DAA. The 95% confidence intervals for the indirect (−879.78, −216.56) and direct (−1233.46, −428.32) effects excluded zero, which further supports partial mediation. Figure 2 presents the standardized path coefficients of the mediation model.

## 4 Discussion

By adapting the debt management game originally developed by Amar et al. [6], the current study aimed to examine whether undergraduate dental students in South Korea exhibit DAA in their repayment decisions and to explore financial literacy as a potential factor that mitigates this bias. The findings revealed that the participants displayed repayment behaviors consistent with DAA, which deviates from the financially optimal strategy by prioritizing small debts over those with high interest rates. Mediation analysis further indicated that financial literacy was negatively associated with DAA and directly and indirectly decreased total remaining debt.

These findings pose a number of implications for the literature on dental education. First, the observed repayment patterns are consistent with those observed for Study 1 in Amar et al. [6], in which undergraduates in the United States also exhibited DAA and ended the game with an average remaining debt of $41,479. The replication of these findings among dental students in South Korea—an academically high‐achieving group—demonstrates that DAA is a robust behavioral bias that persists across educational contexts and disciplines. Importantly, this contribution differs from prior studies on financial literacy and student debt in the health professions, which have largely conceptualized debt as a psychological burden or career constraint based on self‐reported survey data [12, 13, 14].

Second, beyond replicating prior findings, this study extends the literature by introducing a quantitative DAA index that allows for finer‐grained analysis of individual differences in repayment bias. This index enabled the examination of the relationships of financial literacy with DAA and total remaining debt, thereby offering a clear explanation for the reason underlying the tendency of financially literate students to carry lower total debt through reduced DAA. Importantly, it will enable future research to investigate the associations between the DAA index and related but underexplored factors, such as the perception that small debts represent easy goals to achieve [27] or the psychological relief derived from fully closing an account entirely [28], thus deepening the current understanding of the mechanisms underlying DAA.

Third, the present study highlights the critical role of financial literacy in the formation of debt repayment behavior. Dental students with high levels of financial literacy were less susceptible to DAA; as a result, they carried lower total remaining debt. This finding implies that financial literacy not only provides essential financial knowledge and skills but also helps protect students from cognitive biases that undermine effective debt management. Recent evidence further suggests that explicitly addressing behavioral biases within financial education can itself enhance financial literacy and reduce bias‐related decision errors [29]. Taken together with prior work documenting unmet financial education needs among health professions students [13, 14], the present findings suggest that dental curricula should move beyond knowledge‐based instruction and incorporate behaviorally informed educational strategies, such as simulations and interactive debt management tasks, which directly target cognitive biases like DAA. This training could decrease the vulnerability of dental students to DAA, strengthen their debt management competencies, and, ultimately, support the long‐term sustainability of dental practices.

Despite these significant findings, this study has its limitations. First, the generalizability of the results may be limited to the South Korean context. For example, in the United States, dental schools accredited by the Commission on Dental Accreditation are required to provide instruction in personal debt management and financial planning as part of their curricula [30]. Furthermore, initiatives such as the Dental Practice Readiness Curriculum further embed financial training in curricula to prepare students for real‐world responsibilities such as the management of student loans and development of business plans [31]. Given structural differences in the integration of financial training into curricula across countries, future studies should conduct cross‐national comparisons to elucidate the mechanism through which educational contexts form the relationship between financial literacy and debt management. Second, future research should broaden the sampling framework to include undergraduate dental students from multiple Korean universities and across all academic years (first through fourth year). Such an expanded approach would help verify the generalizability of DAA and further clarify the role of financial literacy in a more diverse student population. Furthermore, rather than relying on a convenience sample, subsequent studies should determine the sample size a priori using a power analysis to improve the precision and reproducibility of results. Third, future research should account for individual financial background factors that may influence debt management behavior. Previous studies have shown that family financial vulnerability and household income are significant predictors of debt attitudes and repayment performance [32, 33]. Building on these findings, subsequent research should examine the effect of financial literacy while controlling for such contextual variables—including personal or familial financial stress, income level, and prior loan experience—to isolate its unique contribution to debt‐related decision‐making. Incorporating these factors would provide a more comprehensive understanding of how financial literacy operates within diverse financial contexts and inform the design of targeted educational interventions for dental students. Fourth, although the game provided a controlled and incentive‐compatible environment, it did not fully reflect the complexity of real‐world debt repayment. Individuals typically make decisions about how to allocate income across multiple accounts for different purposes—such as spending, saving, and investing—rather than dedicating it entirely to debt repayment. Future research should test DAA under conditions that allow for such allocation choices to enhance ecological validity. Fifth, this study used a cross‐sectional design for data collection. This design may have limited inferences regarding the causality of variables. Thus, future studies should also consider using a longitudinal design to capture causal relationships among variables in a comprehensive manner.

## 5 Conclusion

The manifestation of DAA even among academically high‐achieving students, such as dental undergraduates, demonstrates its robustness, and financial literacy emerges as a critical factor that improves debt management by mitigating this bias. To enhance financial literacy effectively, dental education should explicitly address behavioral biases within financial training and promote experiential learning environments where students can recognize and reflect on these biases through computer‐based simulations and interactive games. Embedding this approach in dental curricula may strengthen students’ financial competencies and better prepare them for future career demands such as clinic startup and practice management.

## Conflicts of Interest

The authors declare no conflicts of interest.

## Supporting information




**Supporting File 1**: jdd70211‐sup‐0001‐SuppMat

## Endnotes

1 Under the Korea Medical Service Act (Article 33, Paragraph 2), only licensed healthcare professionals (e.g., dentists) or non‐profit / public institutional entities are eligible to establish and operate medical institutions. As a result, direct establishment or financing of a dental clinic by private investors, corporations, or general capital—including government‐affiliated investment entities—is effectively prohibited.

2 For further information, Cronbach's alpha for the adapted 15‐item version is 0.50 in this study. Prior psychometric research suggests that alpha primarily reflects reliability for homogeneous constructs and may underestimate it for heterogeneous constructs like knowledge. Thus, fixed cutoff values for the alphas of knowledge tests are inappropriate [17, 18, 19, 20]. When such measures demonstrate content validity and theoretical coherence, lower alpha values do not constitute a major impediment to their interpretability or use [17, 18, 19, 20]. Notably, the financial literacy measure used here has been shown to possess strong content validity, as it comprehensively covers both basic and advanced domains of financial knowledge and consistently predicts real‐world financial behaviors such as retirement planning and stock market participation [15, 16, 23].

3 As participants were required to use their entire available budget for debt repayment in each round, the checking account balance was necessarily zero at the end of the game.

4 As with Amar et al.’s study, we assigned a value of 26 to debts that remained open at the end of the game.
