# Supplementary material for: Why Financial Literacy Matters for Dental Students: Reducing Debt Account Aversion to Improve Debt Management
Source: J Dent Educ. 2026 Apr 10;90(8):1237–44. doi: 10.1002/jdd.70211 (PMC13489184; doi:10.1002/jdd.70211)
Supplement: Supplementary file 1 [file JDD-90-1237-s001.docx]

Supporting information

1. Financial Literacy

(a) Basic literacy questions

Q1. *Numeracy*: Suppose you had $100 in a savings account and the interest rate was 2% per year. After 5 years how much do you think you would have in the account if you left the money to grow?

(i) More than $102; (ii) Exactly $102; (iii) Less than $102; (iv) Do not know.

Q2. *Interest compounding*: Suppose you had $100 in a savings account and the interest rate is 20% per year and you never withdraw money or interest payments. After 5 years, how much would you have on this account in total?

(i) More than $200; (ii) Exactly $200; (iii) Less than $200; (iv) Do not know.

Q3. *Inflation*: Imagine that the interest rate on your savings account was 1% per year and inflation was 2% per year. After 1 year, how much would you be able to buy with the money in this account?

(i) More than today; (ii) exactly the same; (iii) less than today; (iv) Do not know.

Q4. *Time value of money*: Assume a friend inherits $10,000 today and his sibling inherits $10,000 three years from now. Who is richer because of the inheritance?

(i) My friend; (ii) His sibling; (iii) They are equally rich; (iv) Do not know.

Q5. *Money illusion*: Suppose that in the year 2015, your income has doubled and prices of all goods have doubled too. In 2010, how much will you be able to buy with your income?

(i) More than today; (ii) The same; (iii) Less than today; (iv) Do not know.

(b) Advanced literacy questions

Q6. *Which of the following statements describes the main function of the stock market?*

(i) The stock market helps to predict stock earnings; (ii) The stock market results in an increase in the prices of stocks; (iii) The stock market brings people who want to buy stocks together with those who want to sell stocks; (iv) None of the above; (v) Do not know.

Q7. *Which of the following statements is correct? If somebody buys the stock of firm B in the stock market:*

(i) He owns a part of firm B; (ii) He has lent money to firm B; (iii) He is liable for firm B‟s debts; (iv) None of the above; (v) Do not know.

Q8. *Which of the following statements is correct?*

(i) Once one invests in a mutual fund, one cannot withdraw the money in the first year; (ii) Mutual funds can invest in several assets, for example invest in both stocks and bonds; (iii) Mutual funds pay a guaranteed rate of return which depends on their past performance; (iv) None of the above; (v) Do not know.

Q9. *Which of the following statements is correct? If somebody buys a bond of firm B:*

(i) He owns a part of firm B; (ii) He has lent money to firm B; (iii) He is liable for firm B‟s debts; (iv) None of the above; (v) Do not know. [*EXCLUDED*]

Q10. *Considering a long time period (for example 10 or 20 years) which asset normally gives the highest return?*

(i) Savings accounts; (ii) Bonds; (iii) Stocks; (iv) Do not know.

Q11. *Normally, which asset displays the highest fluctuations over time?*

(i) Savings accounts; (ii) Bonds; (iii) Stocks; (iv) Do not know.

Q12. *When an investor spreads his money among different assets, does the risk of losing money:*

(i) Increase; (ii) Decrease; (iii) Stay the same; (iv) Do not know.

Q13*. If you buy a 10-year bond, it means you cannot sell it after 5 years without incurring a major penalty. True or false?*

(i) True; (ii) False; (iii) Do not know.

Q14. *Stocks are normally riskier than bonds. True or false?*

(i) True; (ii) False; (iii) Do not know.

Q15. *Buying a company stock usually provides a safer return than a stock mutual fund. True or false?*

(i) True; (ii) False; (iii) Do not know.

Q16. *If the interest rate falls, what should happen to bond prices?*

(i) Rise; (ii) Fall; (iii) Stay the same; (iv) None of the above; (v) Do not know.

2. Initial Screen of Debt Management Game

3. Final Screen of Debt Management Game for a Financially Optimal Player

4. Final Screen of Debt Management Game for a Debt Account Averse Player
